# 6PPDQ Promotes Cutaneous Squamous Cell Carcinoma Growth with PI3K-Akt/MMP9 Activation: Evidence from Integrated Network Toxicology and Experimental Investigation

**DOI:** 10.3390/ijms27188304

**Published:** 2026-09-18

**Authors:** Dekun Song, Yulu Chen, Yue Wu, Yuhao Wu, Lunhui Chang, Xuan Liu, Xiaoxiang Xu, Guorong Yan, Guolong Zhang

**Affiliations:** 1 Department of Phototherapy, Shanghai Skin Disease Hospital, School of Medicine, Tongji University, Shanghai 200443, China; 2 Skin Cancer Center, Shanghai Skin Disease Hospital, School of Medicine, Tongji University, Shanghai 200443, China; 3 Institute of Photomedicine, School of Medicine, Tongji University, Shanghai 200443, China; 4 School of Astronomy and Space Science, Nanjing University, Nanjing 210023, China; 5 Department of Pathology, Shanghai Skin Disease Hospital, School of Medicine, Tongji University, Shanghai 200443, China

**Keywords:** cutaneous squamous cell carcinoma, 6PPD, 6PPDQ, network toxicology, machine learning, molecular docking, molecular dynamics simulation

## Abstract

N-(1,3-dimethylbutyl)-N’-phenyl-p-phenylenediamine (6PPD) and its transformation product 6PPD-quinone (6PPDQ) are emerging tire-derived environmental contaminants, showing a potential tumor-promoting effect. However, their relevance to cutaneous squamous cell carcinoma (cSCC) remains unclear. Here, we used network toxicology and experimental validation to investigate their potential involvement in cSCC progression. A total of 91 candidate genes were identified, and enrichment analysis revealed their participation in oxidative stress response, epithelial proliferation, extracellular matrix degradation, and oncogenic pathways, including PI3K-Akt, MAPK, and IL-17 pathways. Machine learning identified six hub genes: *MMP1*, *MMP9*, *RECK*, *LGALS3*, *GSTP1*, and *FOXM1*. Immune profiling and single-cell RNA-sequencing analysis linked hub-gene expression patterns and tumor microenvironment features, with an emphasis on macrophages, T cells, and keratinocytes. Molecular docking prioritized MMP9 among the six candidate proteins, and molecular dynamics simulations characterized predicted MMP9-ligand complexes. Furthermore, 6PPDQ was found to promote cSCC cell proliferation and tumor growth in both in vitro and in vivo experiments. At the molecular level, 6PPDQ exposure was accompanied by increased PI3K p110α expression, elevated p-AKT/AKT ratio, and MMP9 upregulation, consistent with the computational predictions. Pharmacological AKT inhibition attenuated MMP9 upregulation and the 6PPDQ-associated growth response, while *MMP9* silencing reduced 6PPDQ-enhanced invasion and wound closure. Overall, this bioinformatics-led exploratory study suggests that 6PPDQ has tumor-promoting potential in experimental cSCC models and proposes PI3K-Akt/MMP9 activation as a preliminary mechanistic hypothesis. These findings provide candidate targets for future mechanistic and environmental risk studies.

## 1. Introduction

N-(1,3-dimethylbutyl)-N′-phenyl-p-phenylenediamine (6PPD) is widely used in tires as an antioxidant and antiozonant [1]. Beyond tires, it is also used in other rubber and consumer-related products, including belts, hoses, cables, footwear, hair dyes, nail polish dyes, and lubricants [2]. 6PPD undergoes oxidative transformation upon reaction with ozone in air and forms 6PPD-quinone (6PPDQ), a derivative with higher reported toxicity and persistence [3]. Humans are exposed to these widely distributed compounds through inhalation, ingestion, and possible dermal contact [4,5], resulting in their detection in blood, urine, cerebrospinal fluid (CSF), and breast milk [6,7]. 6PPD/6PPDQ have been reported to induce multi-organ damage (liver, kidney, lung), neurotoxicity, and reproductive toxicity [8,9,10]. Recent evidence also suggests their potential relevance to tumor-promoting processes. Specifically, 6PPD/6PPDQ could form DNA adducts in mammals [11] and induce marked oxidative stress by raising intracellular reactive oxygen species (ROS) and depleting glutathione [12]. Moreover, 6PPD/6PPDQ can induce inflammatory responses in multiple tissues. They induce macrophage-driven pulmonary fibrosis [13], upregulate brain inflammatory factors via HTR2A suppression [10], and provoke intestinal inflammation by epithelial barrier disruption [14]. These findings suggest biological effects relevant to tumor promotion. Therefore, the potential role of 6PPD/6PPDQ in cancer-related processes deserves further study.

Cutaneous squamous cell carcinoma (cSCC) is the second most common form of skin cancer, whose reported incidence has increased by 50–200% over the past 30 years [15]. While UV exposure remains the primary risk factor, environmental toxicants such as arsenic, polycyclic aromatic hydrocarbons (PAHs), and microplastics are also associated with cSCC carcinogenesis [16,17,18]. Direct evidence comes from the standard two-step DMBA/TPA mouse model of cSCC, where 7,12-dimethylbenz[a]anthracene (DMBA) acts as the tumor initiator and 12-O-tetradecanoylphorbol-13-acetate (TPA) as the tumor promoter; their sequential application induces cSCC in mice [19]. While 6PPD and 6PPDQ are environmentally widespread and have been linked to skin-related biological effects [20,21], their potential involvement in cSCC progression remains unexplored.

In this study, we integrated a computational framework with biological experiments to explore the involvement of 6PPDQ in cSCC progression. Through network toxicology and machine learning, we identified six hub genes (*MMP1*, *MMP9*, *RECK*, *LGALS3*, *GSTP1*, and *FOXM1*). Given its environmental relevance and higher predicted toxicity, 6PPDQ was prioritized for experimental validation. Functional and pathway-intervention experiments in vitro and in vivo showed that 6PPDQ promoted cSCC cell proliferation and tumor growth, accompanied by PI3K-Akt pathway activation and MMP9 upregulation. Our results provide a novel molecular perspective on the potential relevance of 6PPDQ to cSCC and identify candidate targets.

## 2. Results

### 2.1. Toxicological Prediction of 6PPD/6PPDQ

**Figure 1:**
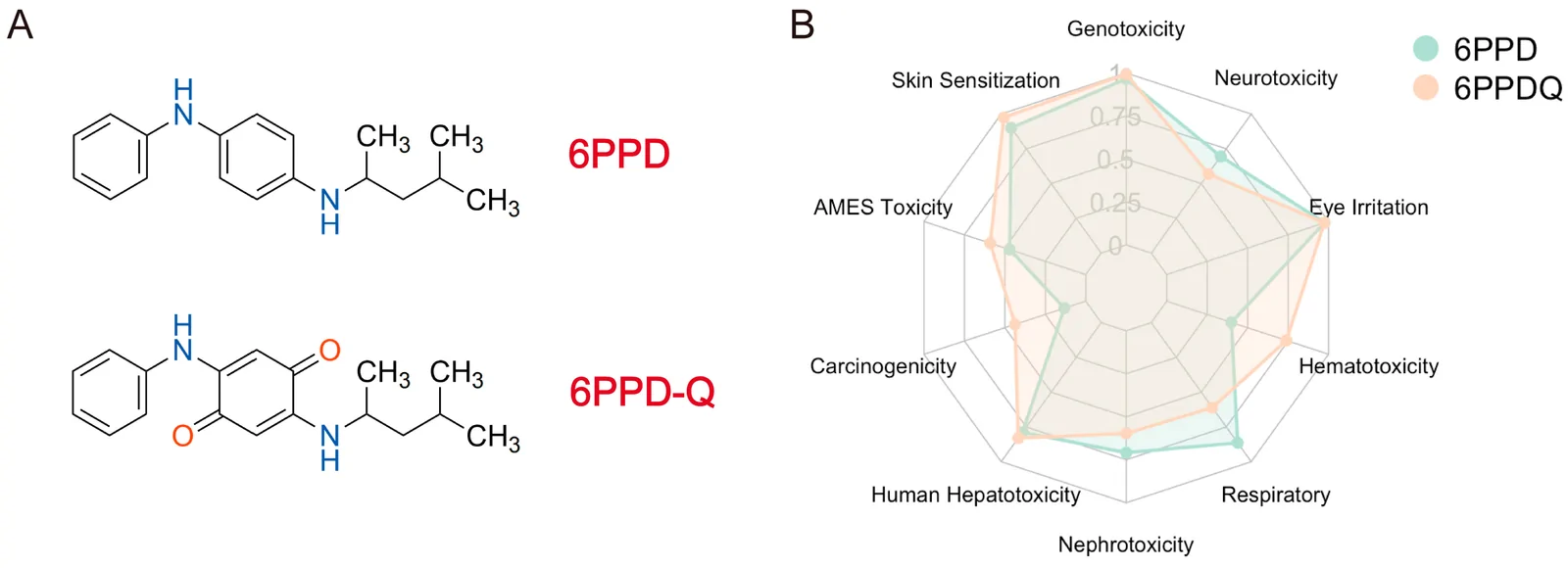
Characterization and Predicted Hazard Assessment of 6PPD/6PPDQ. (**A**) Chemical structure of 6PPD and 6PPDQ; (**B**) predicted toxicity classification of 6PPD and 6PPDQ. The radar chart represents the predicted probability of 6PPD (green) and 6PPDQ (orange) being toxic across ten endpoints, with values ranging from 0 to 1. The classification threshold was 0.5.

The molecular structures of 6PPD/6PPDQ were retrieved from the PubChem database (Figure 1A). A toxicological assessment was conducted using the ADMETlab 3.0 platform (Figure 1B). Compared to 6PPD, 6PPDQ exhibited higher predicted scores for skin sensitization (0.975 vs. 0.901), carcinogenicity (0.437 vs. 0.133), and genotoxicity (0.994 vs. 0.966).

### 2.2. Identification of cSCC-Related Genes

**Figure 2:**
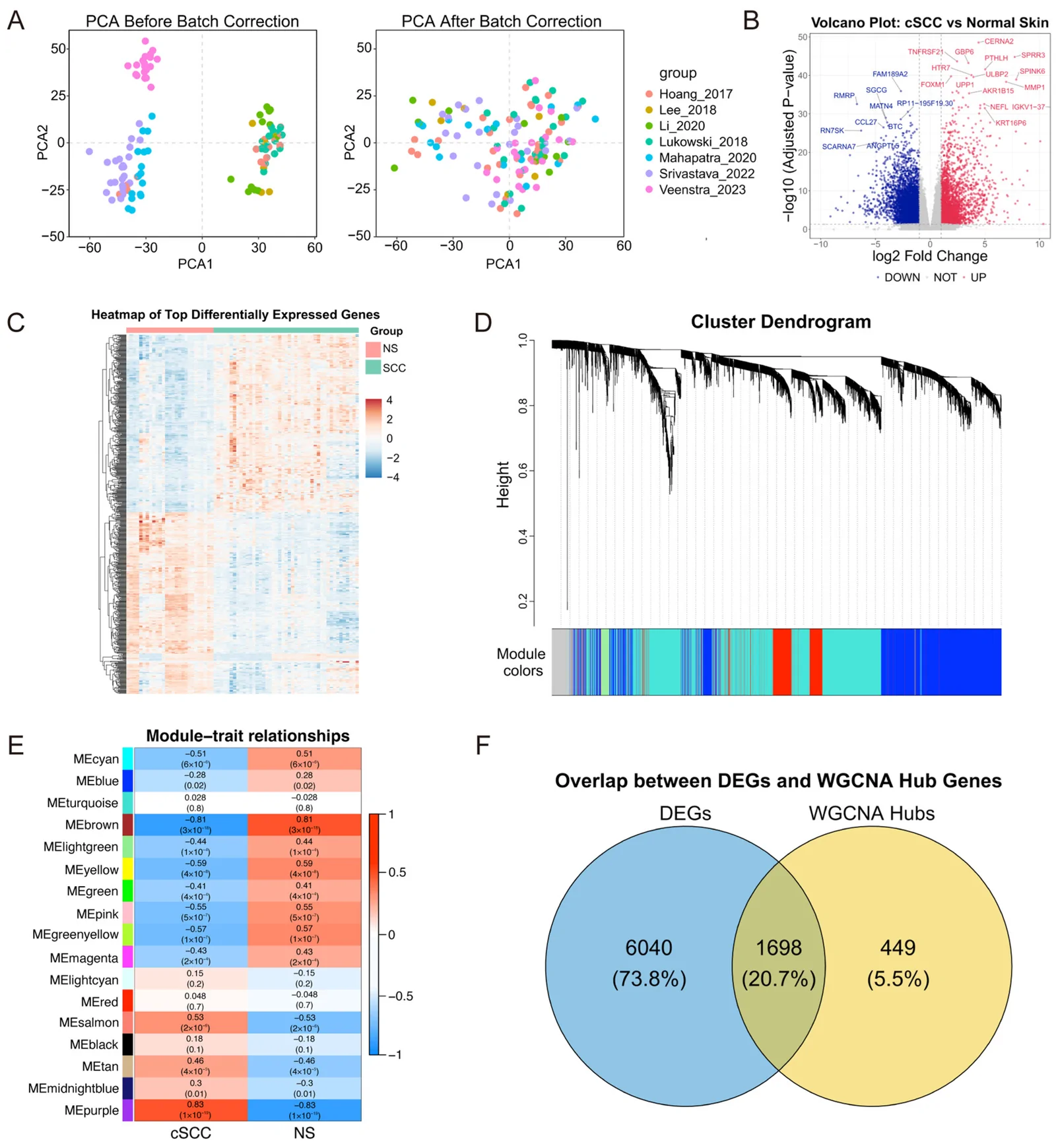
Identification of cSCC-associated target genes. (**A**) PCA plot of samples before and after batch correction, demonstrating improved mixing and reduction in batch effects. (**B**) Volcano plot of DEGs between cSCC and normal skin samples (red: upregulated genes, blue: downregulated genes). (**C**) Heatmap of the top differentially expressed genes across cSCC and normal samples (red: upregulated genes, blue: downregulated genes). (**D**) Gene dendrogram and module assignment from WGCNA. The lower bar displays co-expression modules, each represented by a different color. (**E**) Module–trait correlation heatmap showing the relationship between identified gene modules and clinical traits (cSCC vs. Normal Skin). Each cell contains the correlation coefficient and *p*-value. (**F**) Venn diagram illustrating the overlap between the identified DEGs and genes from significant WGCNA hub modules.

Seven independent cSCC datasets were merged into a training cohort. Batch adjustment substantially reduced study-associated variation, as demonstrated by principal component analysis (PCA; Figure 2A). A total of 7738 DEGs were identified between cSCC and normal skin (NS) samples (Figure 2B), with top DEGs displayed by hierarchical clustering (Figure 2C). For Weighted Gene Co-expression Network Analysis (WGCNA), a soft-thresholding power (β) of 12 was chosen to satisfy the scale-free network topology (R^2^ > 0.8). Multiple gene modules correlated with the cSCC phenotype (*p* < 0.05) were identified (Figure 2D) and detailed by the module–trait correlation heatmap (Figure 2E). By combining the identified DEGs and WGCNA module signatures, we obtained 8187 genes that constitute the GEO-derived cSCC targets (Figure 2F).

### 2.3. Target Gene Prediction and Functional Analysis Between 6PPD/6PPDQ and cSCC

**Figure 3:**
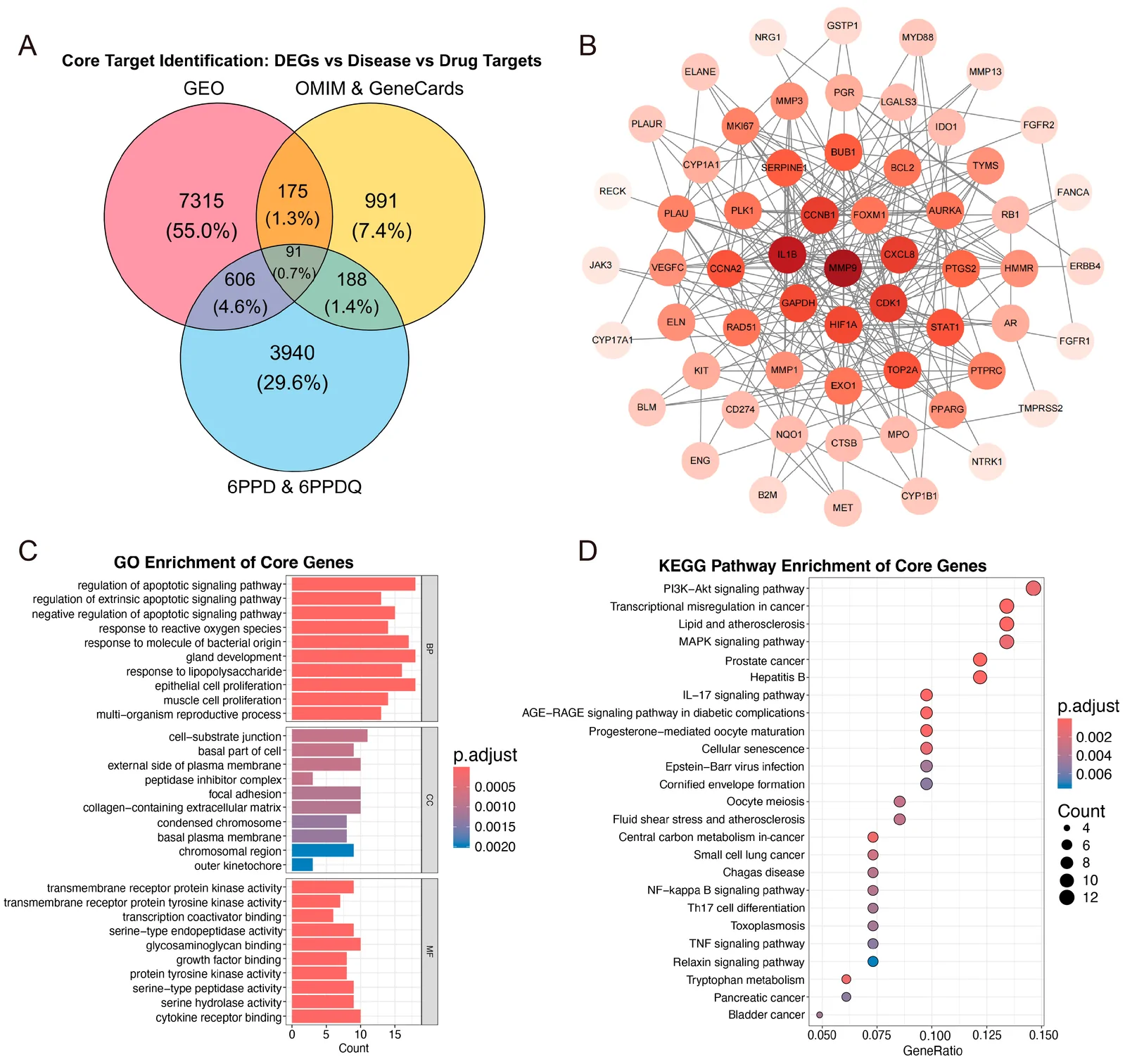
Identification and functional annotation of candidate genes in the toxicant-disease network. (**A**) Venn diagram identifying 91 overlapping candidate genes at the intersection of three gene sets: cSCC-related genes from GEO datasets, disease-associated genes from OMIM and GeneCards, and predicted targets of 6PPD and 6PPDQ. (**B**) PPI network of the 91 candidate genes. Node color is scaled by degree, with darker red indicating higher connectivity. (**C**) GO enrichment analysis of the candidate genes. The bar plot displays the top enriched terms. Bar color represents the adjusted *p*-value. (**D**) KEGG pathway enrichment analysis of the candidate genes. Dot size corresponds to the number of enriched genes, and the color scale represents the adjusted *p*-value.

We used four complementary databases to predict potential protein targets. After merging the predicted results and removing duplicates, 4825 protein targets were obtained for 6PPD/6PPDQ. After intersecting GEO-derived genes with genes retrieved from the OMIM and GeneCards databases, we ultimately identified 266 cSCC-related genes. Then, the intersection of predicted cSCC targets and 6PPD/6PPDQ targets yielded 91 overlapping candidate genes (Figure 3A). The identified 91-gene overlap was substantially greater than the 17.4 expected by chance, with consistent support from 100,000 fixed-size randomizations. protein–protein interaction (PPI) network analysis identified MMP9 as the central hub (node degree = 21; Figure 3B). Gene Ontology (GO) enrichment (Figure 3C) indicated that the 91 genes were enriched in: (i) biological processes involving ROS response, apoptosis, and epithelial proliferation; (ii) cellular components including cell–substrate junctions and extracellular matrix (ECM); and (iii) molecular functions involving receptor kinase and serine-type endopeptidase activities. Enriched Kyoto Encyclopedia of Genes and Genomes (KEGG) pathways included PI3K-Akt, MAPK, IL-17, TNF, and transcriptional misregulation in cancer (Figure 3D). These pathways are broadly interconnected, with PI3K-Akt, MAPK, and IL-17 signaling converging on shared proliferative, inflammatory, and ECM-remodeling processes [22,23].

Further classification revealed 34 6PPD-specific targets, 10 6PPDQ-specific targets, and 47 shared targets (Figure S1A,B), supporting their overlapping yet distinct functional annotations (Figure S1C–F). For 6PPD-specific targets, GO terms were mainly enriched in responses to toxic substances and peptidase inhibitor complex (Figure S1C), with KEGG pathways enriched in lipid and atherosclerosis, and the FoxO signaling pathway (Figure S1D). In contrast, 6PPDQ-specific targets were linked to epithelial cell proliferation, ameboidal-type cell migration, and cell adhesion-related protein complex (Figure S1E), with KEGG pathways focused on human papillomavirus infection, Ras signaling pathway, and chemical carcinogenesis-related pathways (Figure S1F).

### 2.4. Prioritization of Candidate Genes Through Integrative Machine Learning

**Figure 4:**
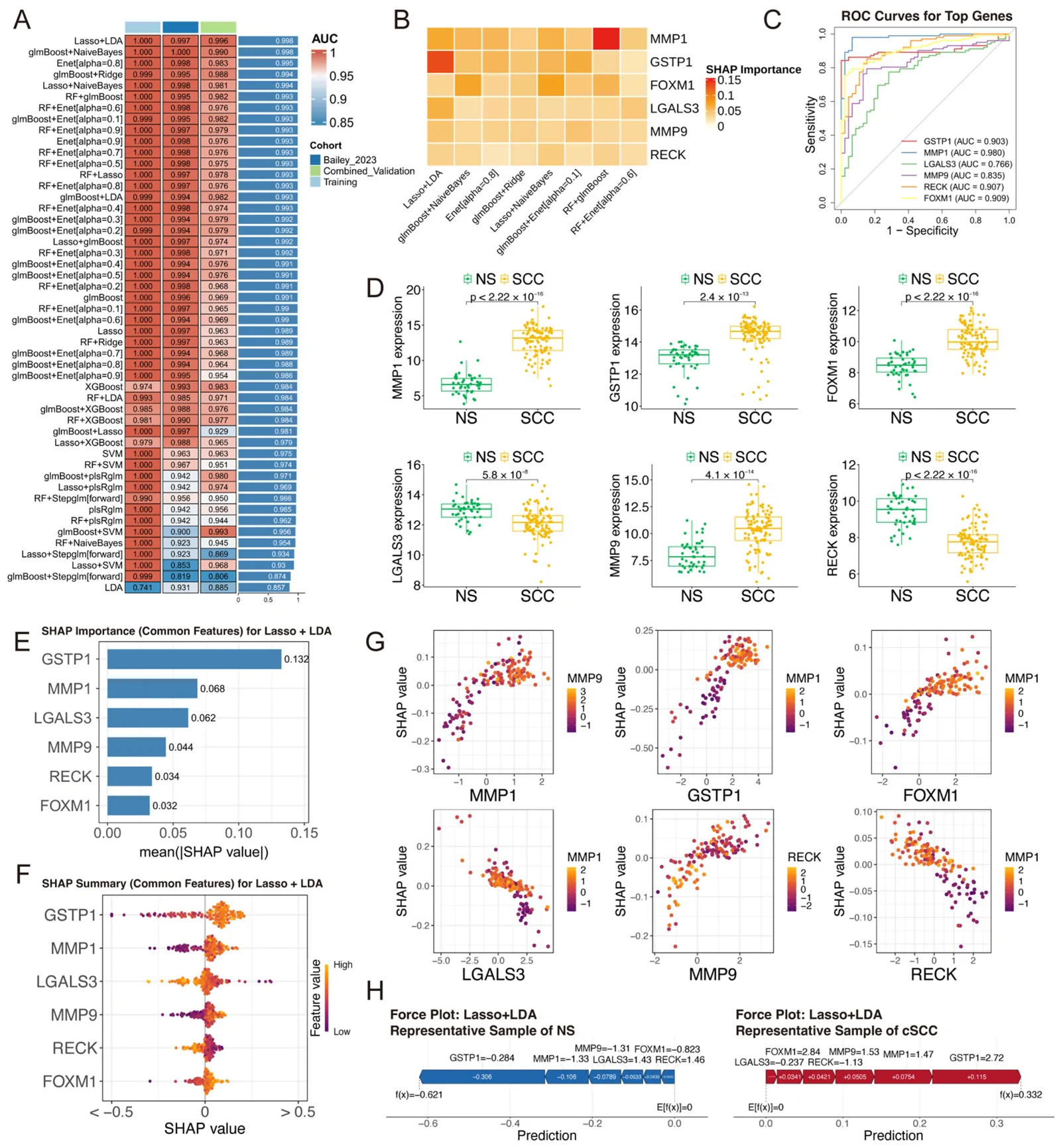
Machine learning-based identification, validation, and interpretation of the candidate diagnostic signature. (**A**) Heatmap displaying the performance (AUC) of machine learning model combinations across the training and two validation cohorts. (**B**) Heatmap of SHAP feature importance for the six hub genes (*MMP1*, *GSTP1*, *FOXM1*, *LGALS3*, *MMP9*, and *RECK*) across top-performing models. These genes represent the common set of features identified from the eight highest-performing model combinations. (**C**) ROC curves for the six identified genes in the validation cohort. AUC indicates predictive performance. (**D**) Boxplots showing the differential expression levels of the six hub genes in NS and cSCC tissues from the validation cohort. (**E**) SHAP feature importance ranking for the top-ranked Lasso + LDA model, with genes ordered by their contribution (measured by mean SHAP value). (**F**,**G**) SHAP value distributions. The summary plot (**F**) illustrates the overall impact and direction of SHAP values for each feature. The dependence plots (**G**) show the gene expression distributions between conditions. (**H**) SHAP force plots of representative samples. Visualization of gene contributions to the model’s prediction for NS and cSCC.

To identify hub genes from the 91 candidate targets, we constructed 101 machine learning models by integrating classical algorithms, and compared the model performance across the training OOF predictions and two validation cohorts (Figure 4A). SHapley Additive exPlanations (SHAP) analysis of the top eight models identified six prioritized genes: *MMP1*, *GSTP1*, *FOXM1*, *LGALS3*, *MMP9*, and *RECK* (Figure 4B). ROC curve analysis showed their diagnostic efficacy (Figure 4C, AUC > 0.75). Expression analysis showed the up-regulation of *MMP1*, *MMP9*, *FOXM1*, and *GSTP1*, and the down-regulation of *RECK* and *LGALS3* in cSCC (Figure 4D). The Lasso + LDA model demonstrated the highest accuracy, which we further interpreted by SHAP analysis (Figure 4E–H). The SHAP summary plot showed high expression of GSTP1, MMP1, MMP9, and FOXM1 correlated with positive SHAP values, whereas high levels of LGALS3 and RECK corresponded to negative values (Figure 4F). SHAP dependence plots further revealed key nonlinear relationships and gene interactions (Figure 4G). Force plots illustrated these patterns at the individual level (Figure 4H).

### 2.5. Immune Infiltration Analysis

**Figure 5:**
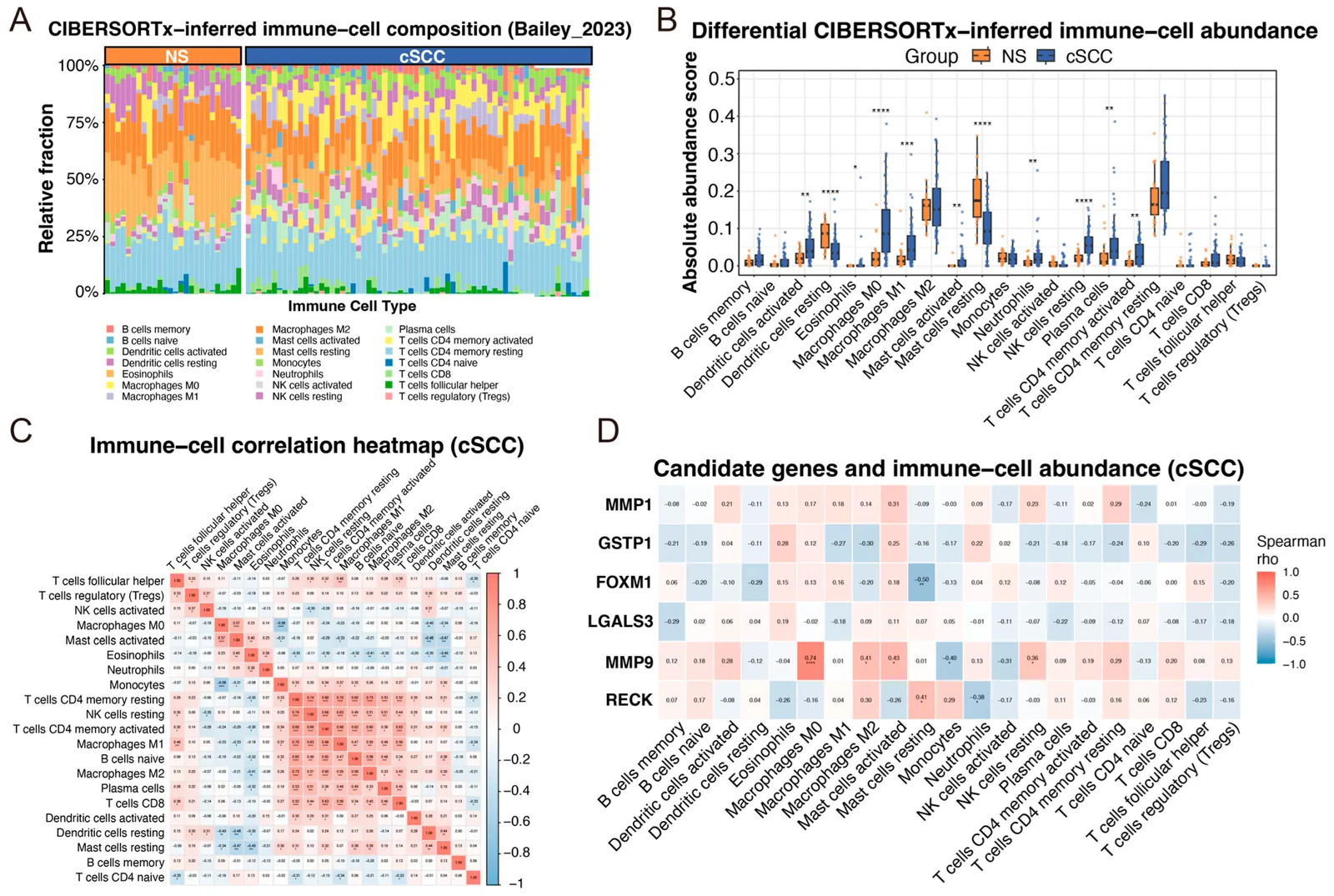
Immune infiltration analysis of cSCC tumor microenvironment. (**A**) Relative immune-cell composition in NS (*n* = 26) and cSCC (*n* = 66) samples from the Bailey_2023 cohort, derived by within-sample normalization of absolute-mode abundance scores. (**B**) CIBERSORTx-inferred absolute immune-cell abundance scores in NS and cSCC. (**C**) Correlation heatmap for 21 immune cell subtypes within the 66 cSCC samples (red: positive correlation; blue: negative correlation). (**D**) Correlation heatmap between six candidate gene expression and immune cell infiltration scores within the 66 cSCC samples (* *p* < 0.05, ** *p* < 0.01, *** *p* < 0.001, **** *p* < 0.0001).

We used CIBERSORTx (https://cibersortx.stanford.edu/) to characterize the immune landscape in NS and cSCC samples from the Bailey_2023 cohort (Figure 5A,B), and identified significant differences across multiple immune-cell populations. In particular, M0 and M1 macrophages and activated memory CD4+ T cells were increased in cSCC, whereas resting mast cells and resting dendritic cells were reduced. Correlation analyses restricted to the 66 cSCC samples revealed distinct associations among immune-cell populations and candidate-gene expression (Figure 5C,D). Notably, *MMP9* showed a strong positive association with CIBERSORTx-inferred M0 macrophage abundance (*R* = 0.74, *FDR* < 0.0001), suggesting that *MMP9* may contribute to shaping a macrophage-associated immune microenvironment in cSCC.

### 2.6. Candidate Gene Expression in Single-Cell RNA Sequencing (scRNA-seq)

**Figure 6:**
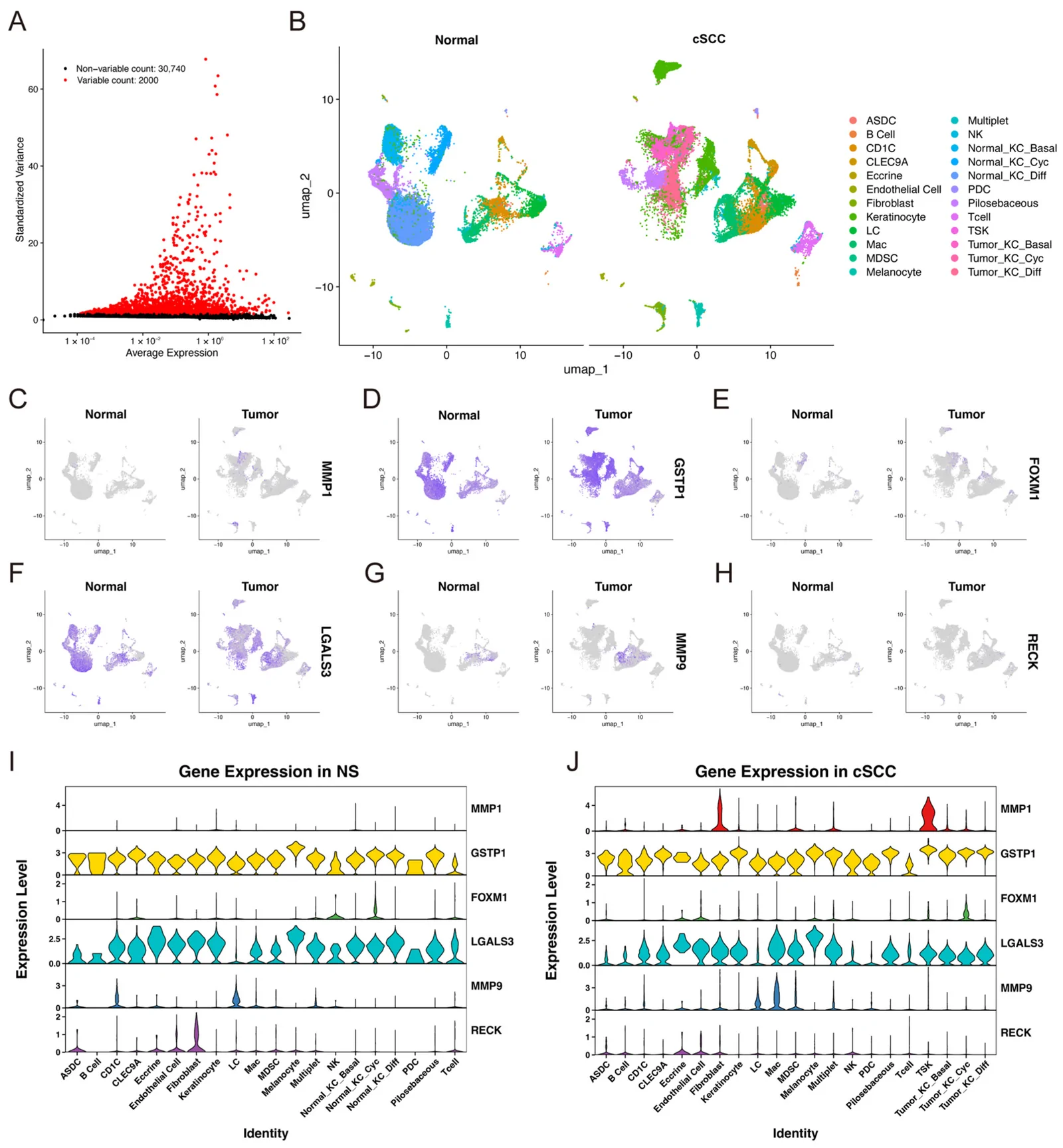
Single-cell transcriptomic analysis of candidate genes. (**A**) Scatter plot of gene variability from the GSE144236 dataset, with the top 2000 variable genes (red points) selected. (**B**) UMAP visualization of single-cell clustering from NS and cSCC, with colors denoting annotated cell types. (**C**–**H**) UMAP feature plots illustrating the expression and spatial distribution of the six candidate genes (*MMP1*, *GSTP1*, *FOXM1*, *LGALS3*, *MMP9*, and *RECK*) in normal and tumor tissues. The color intensity corresponds to the gene expression level. Purple indicates higher gene expression, whereas gray indicates low or undetectable expression. (**I,J**) Violin plots displaying the expression distribution of the six candidate genes across identified cell subtypes in NS and cSCC. TSK: tumor-specific keratinocyte; KC: keratinocyte (Diff, differentiating; Cyc, cycling; Basal, basal); Mac: macrophage; MDSC: myeloid-derived suppressor cell; LC: Langerhans cell; PDC/ASDC/CLEC9A/CD1C, dendritic cell subtypes; NK: natural killer cell.

To further investigate the expression of these candidate genes, we analyzed the public cSCC scRNA-seq dataset (GSE144236) based on the 2000 most variable genes (Figure 6A). A total of 23 cell types were identified and uniform manifold approximation and projection (UMAP) revealed distinct cellular clustering of NS and cSCC tissues (Figure 6B). The six candidate genes showed specific expression patterns (Figure 6C–H), which were further visualized by violin plots (Figure 6I,J). *MMP1* was mainly expressed in tumor-specific keratinocytes (TSKs) and fibroblasts, while *MMP9* was expressed in macrophages (Macs), Langerhans cells (LCs), myeloid-derived suppressor cells (MDSCs) and a subset of TSKs. These expression patterns may be relevant to matrix remodeling in cSCC. *RECK* was highly expressed in fibroblasts and endothelial cells of NS and associated with stromal-mediated suppression of tissue remodeling. *FOXM1* was specifically expressed in tumor-proliferating keratinocytes, consistent with its role of driving cell cycle progression. *GSTP1* and *LGALS3* were present in most cell types. Notably, *GSTP1* showed higher expression in tumor keratinocytes, indicating activated antioxidant defense, whereas *LGALS3* showed lower expression, suggesting functional loss during carcinogenesis.

### 2.7. Molecular Docking of Candidate Targets

**Figure 7:**
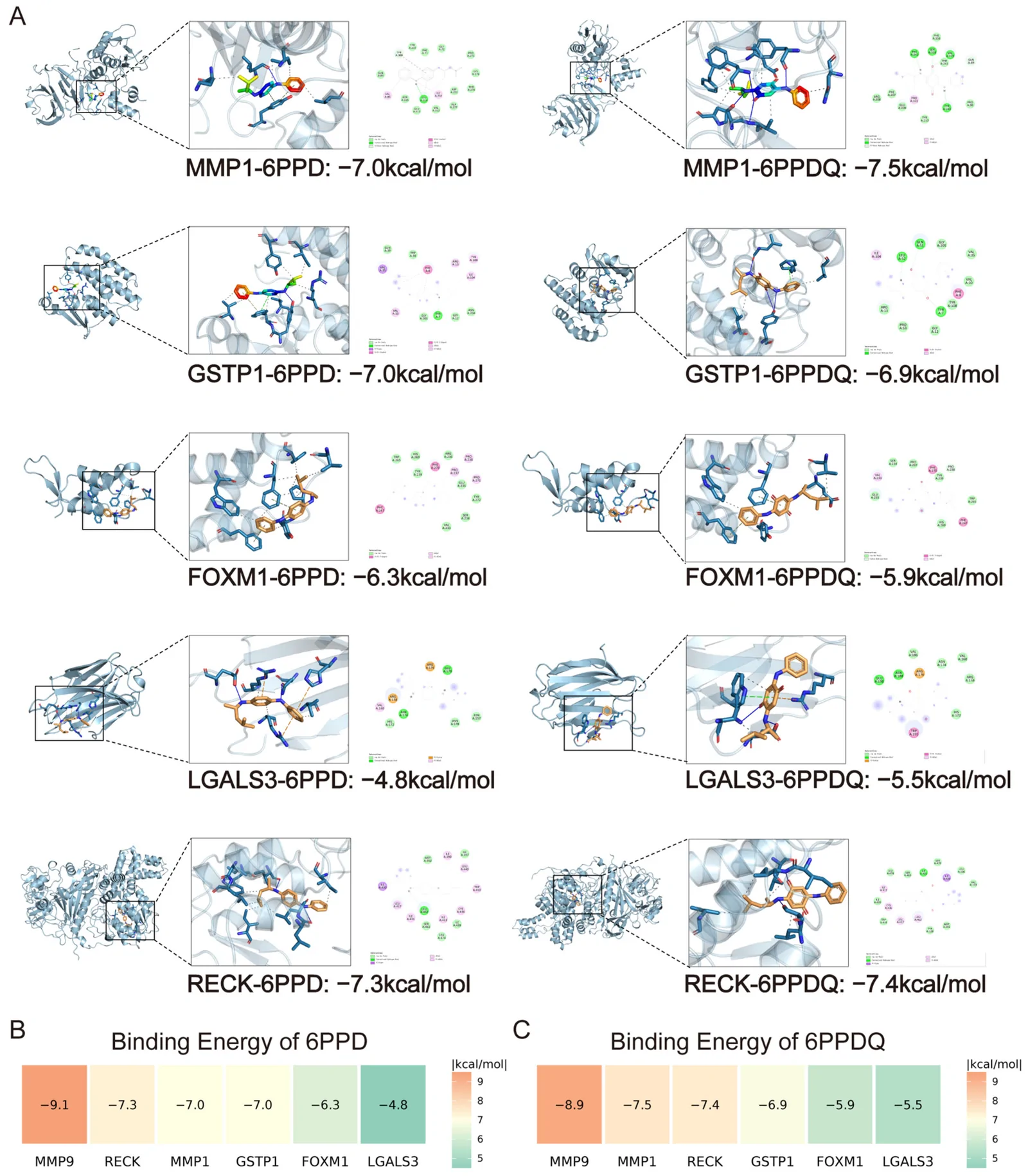
Molecular docking of 6PPD and 6PPDQ with the six proteins. (**A**) Docking poses for the complexes of 6PPD and 6PPDQ with MMP1, GSTP1, FOXM1, LGALS3, MMP9, and RECK, with binding energies indicated. (**B,C**) Heatmaps summarizing the predicted binding energies (kcal/mol) for the interactions of (**B**) 6PPD and (**C**) 6PPDQ with the six proteins.

Molecular docking was performed to predict potential binding modes and docking scores between 6PPD/6PPDQ and six proteins. A binding energy value of <−5.0 kcal/mol suggests favorable predicted binding, while <−7.0 kcal/mol indicates a strong affinity. Results suggested that 6PPD/6PPDQ may form favorable predicted complexes with most of the examined proteins (Figure 7A–C). Notably, the strongest binding was observed for MMP9 with both 6PPD and 6PPDQ (−9.1 and −8.9 kcal/mol), supporting its exploratory prioritization as a candidate target.

### 2.8. Molecular Dynamics (MD) Analysis of MMP9 Complexes

**Figure 8:**
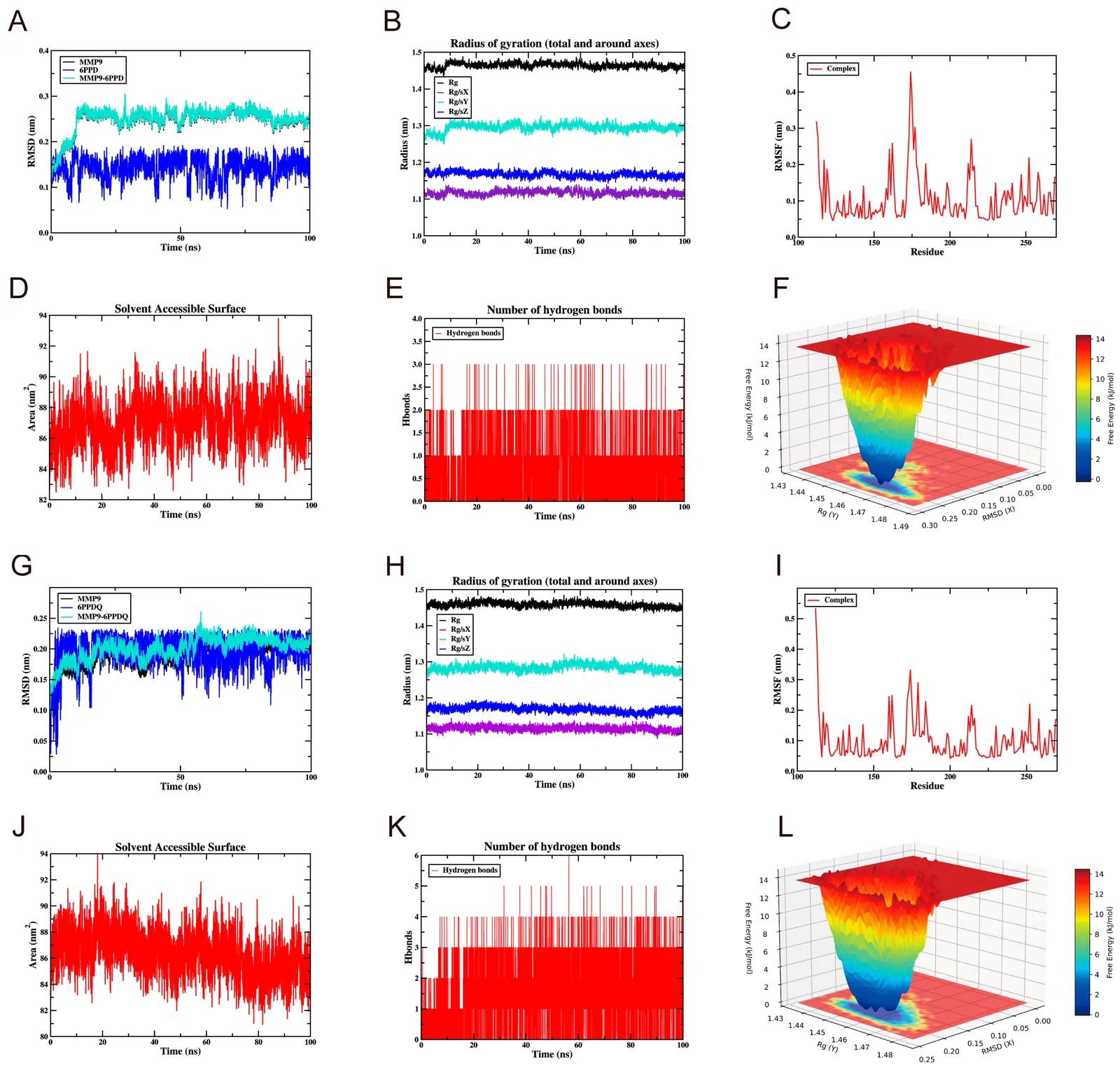
MD simulation of 6PPD/6PPDQ with the key target MMP9. (**A–F**) Analysis of the 100 ns simulation trajectory for the 6PPD-MMP9 complex: (**A**) RMSD of the protein backbone and ligand; (**B**) Rg of the complex; (**C**) RMSF per residue of the protein; (**D**) SASA of the complex; (**E**) number of hydrogen bonds formed between the ligand and protein over time; (**F**) FEL derived from RMSD and Rg parameters; (**G–L**) corresponding analyses for the 6PPDQ-MMP9 complex: (**G**) RMSD, (**H**) Rg, (**I**) RMSF, (**J**) SASA, (**K**) number of hydrogen bonds, and (**L**) FEL.

MMP9 was prioritized for MD simulations based on its central PPI position, favorable docking performance, and significant association with cSCC invasion. The 6PPD–MMP9 and 6PPDQ–MMP9 complexes were then simulated to assess their conformational behavior. Both complexes showed relatively stable structural metrics within the sampled trajectories. Their RMSD values rapidly leveled off and remained below 0.3 nm (Figure 8A,G). Stable and comparable Rg values (1.4–1.5 nm; Figure 8B,H) and SASA values (82–92 nm^2^; Figure 8D,J) indicated compact binding interfaces without structural unfolding. The RMSF (Figure 8C,I) values showed minimal and similar fluctuation patterns in both systems, with moderate flexibility on active regions (e.g., residues 160–180). FEL analysis further identified low-energy basins within the sampled conformational space (Figure 8F,L). Despite the comparable structural stability, key differences existed in their interaction modes. 6PPDQ-MMP9 (Figure 8K) formed more hydrogen bonds (2–5) than 6PPD-MMP9 (1–3, Figure 8E), yet MM-PBSA analysis (Table 1) showed stronger estimated binding free energy of 6PPD-MMP9 than 6PPDQ-MMP9 (ΔGbind = −213.98 kJ/mol vs. −198.74 kJ/mol). This could be attributed to the higher polar solvation penalty of 6PPDQ than 6PPD (ΔGPB = 63.85 kJ/mol vs. 47.01 kJ/mol).

### 2.9. 6PPDQ Promotes cSCC Cell Proliferation, Migration and Invasion via PI3K-Akt-MMP9 Axis In Vitro

**Figure 9:**
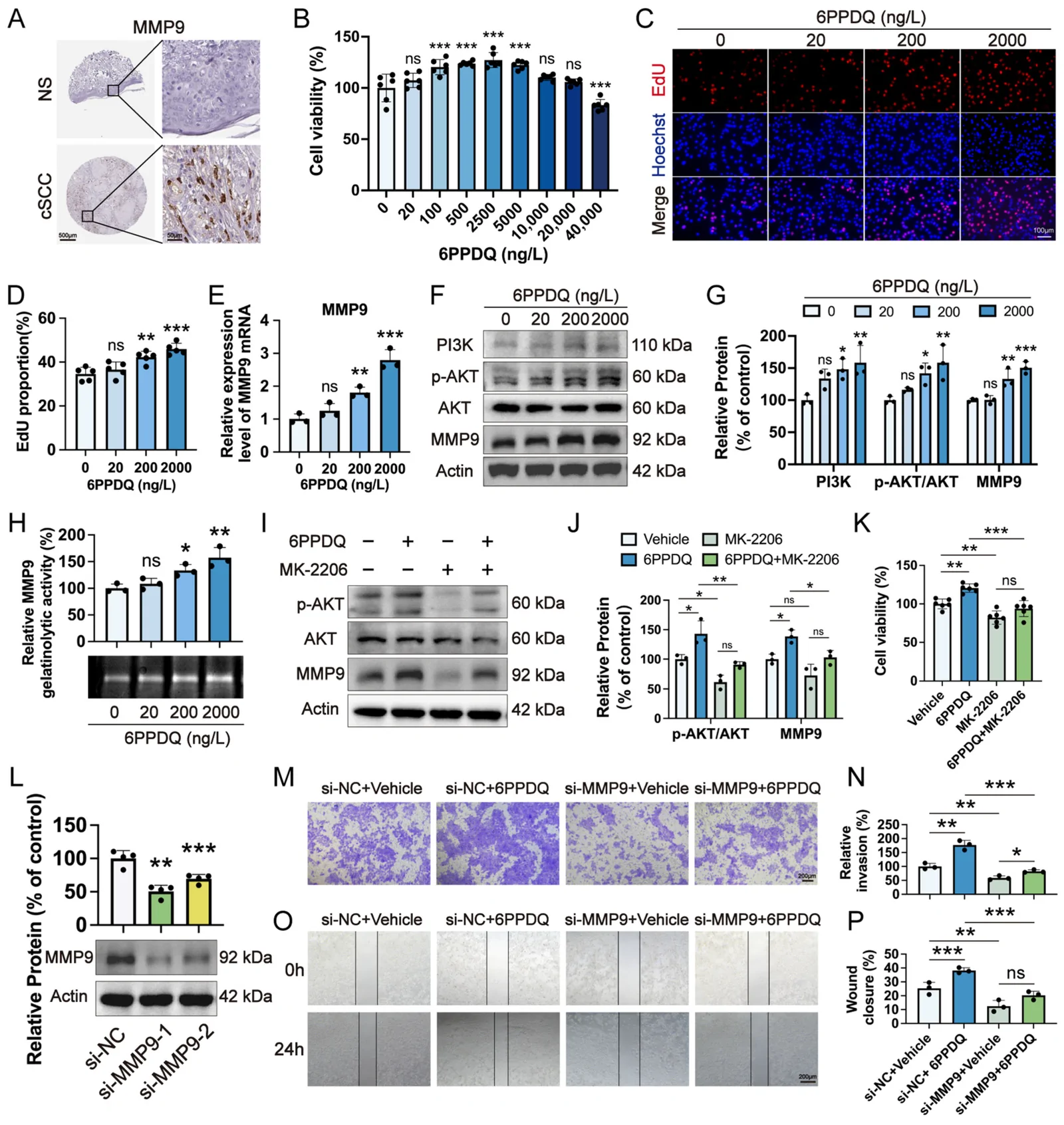
6PPDQ activates PI3K-Akt/MMP9 signaling and promotes proliferative and invasive phenotypes in A431 cells. (**A**) Representative IHC images of MMP9 in normal skin (patient ID: 4093) and cSCC tissues (patient ID: 4809) from the HPA database. (**B**) Viability of A431 cells following 24-h treatment with 6PPDQ. (**C**,**D**) Representative images (**C**) and quantification (**D**) of EdU incorporation of A431 cells after 24-h 6PPDQ exposure. (**E**) Relative *MMP9* mRNA expression in A431 cells. (**F**,**G**) Western blot (**F**) and corresponding quantification (**G**) of PI3K, p-AKT (Ser473)/AKT, and MMP9 in A431 cells. (**H**) Gelatin zymography of conditioned media and quantification of MMP9 gelatinolytic activity following 6PPDQ exposure. (**I**,**J**) Western blot (**I**) and corresponding quantification (**J**) of p-AKT (Ser473)/AKT and MMP9 following treatment with 6PPDQ and/or MK-2206. (**K**) CCK-8 analysis under the same treatments. (**L**) Western blot validation and quantification of *MMP9* knockdown by two independent siRNAs. (**M**,**N**) Representative Matrigel invasion images (**M**) and corresponding quantification (**N**) after si-MMP9 and 6PPDQ treatment. (**O**,**P**) Representative wound-healing images at 0 and 24 h (**O**) and corresponding quantification (**P**)**.** Data are mean ± SD. Sample sizes (n per group): CCK-8, 6 replicates; EdU, 5 replicates; qRT-PCR, Western blot, gelatin zymography, wound-healing, and invasion assays; ≥3 independent biological replicates. Scale bars: (**A**), 500 μm (left) and 50 μm (right); (**C**), 100 μm; (**M**,**O**), 200 μm. * *p* < 0.05, ** *p* < 0.01, *** *p* < 0.001; ns, not significant.

Representative Human Protein Atlas (HPA) IHC images showed stronger MMP9 staining in cSCC compared to normal skin section (Figure 9A). To evaluate the biological impact of 6PPDQ on cSCC, CCK-8 assays were performed on A431 cells. Following 24-h exposure, 6PPDQ exhibited a biphasic effect: concentrations from 100 to 5000 ng/L promoted cell proliferation, whereas the CCK-8 signal decreased at 40,000 ng/L (Figure 9B). The pro-proliferative effect of 6PPDQ at 20, 200, and 2000 ng/L was further evaluated by EdU incorporation assays, showing a dose-dependent increase in proliferating cells (Figure 9C,D).

We further analyzed A431 cells exposed to 6PPDQ (20, 200, and 2000 ng/L) for 24 h. Consistent with the KEGG analysis results, Western blot revealed a dose-dependent increase in PI3K p110α expression and the p-AKT/AKT ratio (Figure 9F,G). Furthermore, MMP9 mRNA (Figure 9E) and protein levels (Figure 9F,G), together with gelatinolytic activity (Figure 9H), increased dose-dependently following 6PPDQ exposure, supporting MMP9 involvement in the cellular response to 6PPDQ. Together, these results indicate that 6PPDQ may promote cSCC cell proliferation by activating the PI3K-Akt pathway and upregulating MMP9.

To examine the effect of AKT inhibition, A431 cells were pretreated with MK-2206 (1 μM) for 1 h and then co-treated with 6PPDQ (2000 ng/L) for 24 h. Compared with 6PPDQ treatment alone, MK-2206 co-treatment reduced the p-AKT/AKT ratio and MMP9 protein expression (Figure 9I,J), together with a lower CCK-8 signal (Figure 9K). These findings support that AKT activation contributes to 6PPDQ-induced MMP9 upregulation and the pro-proliferative response.

To further define the functional role of MMP9, we performed siRNA-mediated *MMP9* knockdown. Both si-MMP9-1 and si-MMP9-2 reduced MMP9 protein expression compared with si-NC (Figure 9L). Because si-MMP9-1 showed greater knockdown efficiency, it was selected for subsequent assays. In si-NC-transfected cells, treatment with 2000 ng/L 6PPDQ for 24 h increased wound closure and Matrigel invasion, whereas *MMP9* knockdown significantly attenuated both effects (Figure 9M–P). These results indicate that MMP9 plays a functional role in 6PPDQ-regulated migration and invasion.

### 2.10. 6PPDQ Accelerates cSCC Tumor Growth with Increased AKT Phosphorylation and MMP9 Expression In Vivo

**Figure 10:**
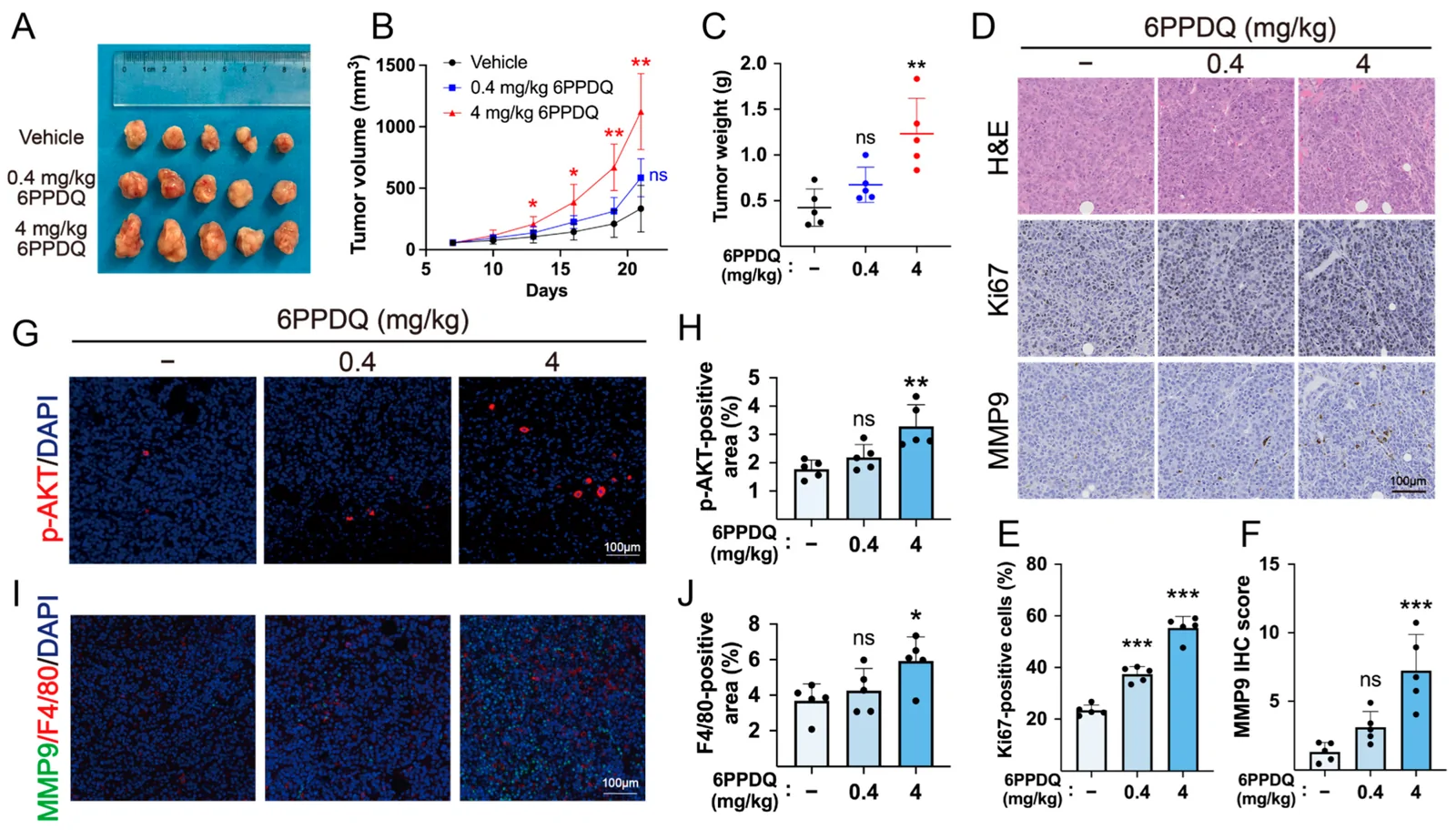
6PPDQ promotes cSCC tumor growth accompanied by AKT activation and MMP9 upregulation in vivo. (**A**–**C**) Representative images of excised tumors (**A**), tumor growth curves (**B**), and final tumor weights (**C**) of SKH-1 mouse tumors treated with 6PPDQ; (**D**–**F**) H&E and IHC staining (Ki67, MMP9) of the tumor tissues (**D**), with corresponding quantitative analysis (**E**,**F**); (**G**,**H**) representative IF images of p-AKT (Ser473)/DAPI (**G**) and quantification (**H**); (**I**,**J**) representative MMP9/F4/80/DAPI double-IF images (**I**) and quantification of F4/80-positive area (**J**). The study timeline was as follows: tumor-cell implantation, day 0; tumor confirmation, group allocation, and first dose, day 7; subsequent dosing every 3 days; and terminal sampling, day 21. Data are mean ± SD; *n* = 5 mice per group. Scale bars: (**D**,**G**,**I**), 100 μm. * *p* < 0.05, ** *p* < 0.01, *** *p* < 0.001; ns, not significant.

We established a subcutaneous cSCC model by co-injecting murine XL50 cells and NIH/3T3 cells into immunocompetent SKH-1 hairless mice. The mice received intraperitoneal 6PPDQ at 0.4 or 4 mg/kg every 3 days. Consistent with previous repeated-exposure mouse studies, 0.4 and 4 mg/kg were used as low- and high-dose levels to evaluate 6PPDQ effects under controlled systemic exposure [8,24,25]. As a reference for human exposure levels, 6PPDQ has been detected in blood and urine at sub-ng/mL to low-ng/mL levels [26], and in environmental matrices such as road dust (ng/g) and airborne particles (pg/m^3^) [27,28]. Given the lack of comparable human skin concentration data and the inability to directly convert cross-matrix exposure levels into intraperitoneal doses, this regimen was used to assess biological effects under defined conditions rather than to model real-world dermal exposure. At 4 mg/kg, 6PPDQ significantly increased tumor volume from day 13 onward and final tumor weight at day 21 compared with the vehicle control group, whereas no significant increase was observed in the 0.4 mg/kg group (Figure 10A–C). H&E staining showed histopathological features consistent with cSCC (Figure 10D). IHC analysis further showed increased Ki67-positive cells in 6PPDQ-treated tumors (Figure 10D,E), while MMP9 expression was significantly elevated in the high-dose 6PPDQ group (Figure 10D,F). In vivo IF further showed increased p-AKT and F4/80-positive areas in the high-dose 6PPDQ group, with F4/80-positive macrophages frequently observed in close spatial proximity to MMP9-positive regions (Figure 10G–J). These results further support that 6PPDQ promotes tumor growth accompanied by increased AKT phosphorylation and MMP9 expression in this experimental cSCC model.

## 3. Discussion

cSCC is the second most common cutaneous malignancy, and its diagnosis, treatment, and prevention are of considerable clinical importance. Current management of cSCC is stratified by risk, with surgery remaining the cornerstone and Mohs micrographic surgery preferred for high-risk cSCC [29,30]. Selected in situ or low-risk lesions may also be treated with shave excision, curettage and electrodesiccation, cryotherapy, photodynamic therapy, or radiotherapy [29,30,31]. For locally advanced or metastatic cSCC, PD-1 blockade is currently the mainstay of systemic therapy, whereas EGFR-targeted therapy and chemotherapy are reserved for selected patients [30,32]. Neoadjuvant immunotherapy is also emerging for resectable high-risk disease [33]. Despite these advances, systemic therapy for cSCC remains limited by low response rates and significant treatment-related toxicity, underscoring the need to explore additional therapeutic targets and molecular mechanisms [32,33,34].

Our study provides integrated computational and experimental evidence suggesting a potential role of 6PPDQ-associated molecular networks in cSCC progression. By integrating multi-omics, machine learning, and structural dynamics, we identified a six-gene candidate signature (*MMP1*, *MMP9*, *RECK*, *LGALS3*, *GSTP1*, and *FOXM1*) with high diagnostic potential. Among these candidates, *MMP9* was further prioritized and experimentally evaluated in vitro and in vivo. Our findings elucidate two convergent toxicological axes—ECM dysregulation and oxidative stress-driven proliferation—through which 6PPDQ may promote tumor aggression.

The first axis involves ECM disruption driven by *MMP1*, *MMP9*, *RECK*, and *LGALS3* dysregulation. Matrix metalloproteinases (MMPs) are critical drivers of tissue remodeling and epithelial-to-mesenchymal transition (EMT), facilitating basement membrane disruption and dermal invasion [35,36]. Consistent with reports on other environmental pollutants (e.g., PM, thirdhand smoke), our functional analysis suggests that 6PPD/6PPDQ likely upregulates *MMP1* and *MMP9* via a similar ROS-mediated inflammatory pathway [37,38]. MMP9 (Gelatinase B), mainly produced by infiltrating inflammatory cells and cSCC tumor cells in invasive edges [36], promotes tumor aggression and keratinocyte migration via ERK signaling [39,40]. The pivotal role of MMP9 for cSCC invasiveness has been confirmed, as direct inhibition via CRISPR-Cas9 significantly reduces cell viability, migration, and the expression of VEGF-A and vimentin [41]. In our study, MMP9 occupies a central position in the PPI network and shows the strongest predicted binding affinity with 6PPD/6PPDQ in molecular docking and MD simulations. Our experiments demonstrated that 6PPDQ dose-dependently elevates MMP9 expression and gelatinolytic activity. AKT inhibition attenuated 6PPDQ-induced MMP9 upregulation and cell proliferation, supporting AKT as an upstream regulator of MMP9. *MMP9* silencing further reduced 6PPDQ-enhanced wound closure and invasion. In vivo, 4 mg/kg 6PPDQ-treated tumors also showed increased p-AKT staining and MMP9 expression. Acting in concert with MMP9, *MMP1* (Collagenase-1) is mainly expressed in TSKs and fibroblasts. MMP1 levels are significantly higher in aggressive cSCC compared with non-aggressive cSCC [42] and positively correlated with depth of invasion and microvascular density [40].

The proteolytic activity of MMPs is further amplified by the concurrent downregulation of RECK (Kazal motifs), a membrane-anchored MMP inhibitor and established tumor suppressor [43]. RECK normally limits MMP9 by inhibiting its catalytic activity and preventing precursor release [44]. This inhibition is clinically relevant: in inflammatory settings, RECK downregulation is linked to MMP9 hypersecretion and enhanced activity [45]. This disrupts ECM integrity and angiogenesis regulation [46], ultimately favoring tumor growth and metastasis.

The expression of *LGALS3* (galectin-3) was also downregulated in cSCC, consistent with previous pathological studies [47]. In head and neck squamous cell carcinoma (HNSCC), the expression of galectin-3 and its reactive ligands is positively correlated with tumor differentiation degree. It also co-localizes with desmosomal proteins and participates in the maintenance of intercellular junctions and mechanical strength [48]. Therefore, the dysregulation of *MMP1/9*, *RECK* and *LGALS3* suggests a potential ECM-remodeling network in cSCC. MMP9 upregulation after 6PPDQ exposure further supports this mechanism.

The second axis includes *GSTP1* and *FOXM1* and centers on the dysregulation of cellular defense and proliferation control. *GSTP1* (glutathione S-transferase P1) is a key Phase II detoxification enzyme involved in cellular antioxidant defense [49] and was upregulated in tumor-specific keratinocytes in our analysis. Its overexpression has also been associated with tumor progression and poor prognosis [50]. It inhibits JNK phosphorylation by modulating the TRAF2-ASK1 interaction, and thereby blocks the apoptosis pathway [51,52]. In lung adenocarcinoma, the hypoxic microenvironment can upregulate GSTP1 via the CaMK2A/NRF2 signaling axis. This maintains the self-renewal ability of cancer stem cells (CSCs) by suppressing excessive intracellular ROS and promoting tumor metastasis and therapy resistance [53].

*FOXM1* (forkhead box protein M1), a cell cycle regulator, was also upregulated in cSCC, mainly in proliferating tumor keratinocytes. This aligns with prior studies [54], which identified FOXM1 as a key transcription factor regulating the self-renewal and proliferation potential of epidermal stem cell. FOXM1 can drive cells to bypass the differentiation-mitosis checkpoints and promote G2/M transition, thereby enhancing proliferative capacity [55]. In addition, it provides pro-survival signals for tumors by activating the PI3K-Akt pathway [56] and inhibiting the FOXO1/RB1 pathway [57], and enhances invasion via MMP2/MMP9 upregulation [58]. Collectively, the upregulation of *GSTP1* and *FOXM1* may contribute to cSCC progression by supporting apoptosis evasion and enhanced proliferative capacity.

This study has several limitations. First, the public cSCC datasets have small sample sizes and inter-study heterogeneity, which may limit the generalizability of this finding. Second, although we validated our findings in cSCC cell and mouse models, the in vitro validation was limited to a single human cSCC line. Responses to 6PPDQ may vary across cSCC models with different genetic and phenotypic backgrounds. Additional cSCC lines, primary human keratinocytes or skin organoids are needed to assess generalizability. The relatively small animal group size and two-dose design limited precise dose–response assessment. In addition, intraperitoneal injection enabled controlled systemic exposure and accurate dosing, but it does not mimic real-world inhalational or dermal exposure, and measured 6PPDQ levels in human skin remain unavailable. Future studies using validated topical exposure models and exposure-relevant dosing are therefore needed. Direct binding between 6PPDQ and MMP9 also remains to be experimentally validated.

Therefore, future studies using validated topical exposure models and exposure-relevant dosing are needed to determine whether dermal 6PPDQ exposure is sufficient to promote cSCC progression. Moreover, real-world epidemiological data quantifying the cSCC risk from 6PPD/6PPDQ exposure remain lacking. Accordingly, the present findings support the tumor-promoting potential of 6PPDQ in experimental cSCC models but cannot establish population-level cancer risk or causality in humans, particularly given potential cross-species differences in exposure routes, metabolism, immune responses, and tumor microenvironment. Future occupational cohort or case–control studies in highly exposed populations are needed to validate these findings. Despite these limitations, this study provides multi-level evidence supporting the tumor-promoting potential of 6PPDQ. Although preliminary, this study may lend support to the important role of MMP9 in cSCC progression, as revealed through the environmental pollutant 6PPDQ, thereby increasing attention to its therapeutic relevance and encouraging further research.

## 4. Materials and Methods

### 4.1. Construction of cSCC Cohorts for Model Training and Validation

A transcriptomic cohort of 220 samples (147 cSCC and 73 normal skin) from 11 studies was derived from the uniformly processed resource established by Bencomo and Lee [59], in which raw RNA-seq data from the included studies were reprocessed using a common STAR/RSEM alignment and quantification workflow. Seven studies were assigned to the training cohort and the remaining four studies to two validation cohorts at the study level (Table S1). Batch effects were adjusted using limma::removeBatchEffect on variance-stabilizing transformation (VST) data within the training set, while condition-related biological variation was retained in the design matrix, with efficacy assessed by PCA. Model performance was then evaluated on two distinct, uncorrected validation cohorts. Model fitting, preprocessing, and within-model feature selection were restricted to the training set.

### 4.2. Identification of cSCC-Associated Genes

DESeq2 was used to screen differentially expressed genes (DEGs) in the training cohort using a ~ study + condition design. Genes with adjusted *p* < 0.05 and |log_2_FC| > 1.0 were retained. Concurrently, we used WGCNA to identify phenotype-correlated hub genes (|r| > 0.4, *p* < 0.05; |MM| > 0.7, |GS| > 0.3) using established parameters (scale-free R^2^ > 0.8, TOM merge height = 0.25, minimum module size = 30). The final list of cSCC-related genes was identified by intersecting the union of DEGs and WGCNA hub genes with reported cSCC-associated genes from the OMIM and GeneCards databases.

### 4.3. Toxicity Profiling and Identification of 6PPD/6PPDQ-Associated Genes

The SMILES sequences and structural data of 6PPD/6PPDQ were downloaded from the PubChem database. Toxicological assessment was performed using the ADMETlab 3.0. Classification scores represent probabilities of a positive result (threshold: 0.5). The predictions were used for preliminary screening. 6PPD/6PPDQ-associated genes were obtained from two distinct evidence sources. Curated chemical–gene associations were retrieved from the Comparative Toxicogenomics Database (CTD), whereas computationally predicted protein-target candidates were obtained from PharmMapper, SwissTargetPrediction, and the Similarity Ensemble Approach (SEA), with *Homo sapiens* specified as the target organism. We merged target lists and removed duplicates to generate a non-redundant set of targets for each compound.

### 4.4. Identification and Statistical Enrichment of Overlapping Candidate Genes

Candidate pathogenic genes were defined as the overlap between cSCC-related genes and predicted 6PPD/6PPDQ targets. This was visualized using a Venn diagram.

### 4.5. Construction of the PPI Network

We analyzed the overlapping genes using STRING and set the organism to *Homo sapiens*. High-confidence interactions (score > 0.7) were retained. The results were analyzed in Cytoscape 3.10.3 for topological characteristics.

### 4.6. Enrichment Analysis

GO and KEGG pathway enrichment analyses were conducted with the clusterProfiler R package 4.10.1. GO terms were examined under biological process (BP), cellular component (CC), and molecular function (MF). Terms or pathways with adjusted *p* values below 0.05 were regarded as significant.

### 4.7. Machine Learning and Model Interpretation

To prioritize a candidate gene signature, 101 predictive models were constructed on the batch-corrected training set using combinations of 11 classical algorithms (LASSO, SVM, Ridge, Random Forest, glmBoost, XGBoost, Elastic Net, Stepglm, plsRglm, LDA, and Naive Bayes). The overlapping targets identified in the preceding analyses constituted an a priori candidate feature pool. Training performance was evaluated using stratified 5-fold out-of-fold (OOF) predictions. Within each outer fold, standardized expression, feature selection and model fitting were in the corresponding training subset, before prediction of the held-out samples.

Feature selection and model fitting were performed using the training set, with validation cohorts kept separate. In two-stage models, the first algorithm selected features and the second constructed the classifier. Models were optimized via 5-fold cross-validation and ranked by the sample-size-weighted mean AUC across cohorts. The eight highest-performing models were interpreted using SHAP, and genes with consistent selection and stable SHAP contributions were retained as the consensus diagnostic signature.

### 4.8. Immune Infiltration Analysis

The immune landscapes of cSCC and NS were characterized using CIBERSORTx with the LM22 signature matrix in absolute mode, with B-mode batch correction, 1000 permutations, and quantile normalization disabled for RNA-seq data. All 92 samples met the deconvolution quality criterion (permutation-derived *p* < 0.05). Differential abundance between groups was assessed by Wilcoxon rank-sum test. Spearman correlation analysis was used to evaluate co-infiltration among immune cell subtypes and their correlations with target gene expression. *p* values from multiple comparisons were adjusted using the Benjamini–Hochberg method.

### 4.9. scRNA-seq Analysis

We performed the descriptive analysis of the processed and annotated scRNA-seq dataset GSE144236 (comprising 48,164 cells from 10 paired cSCC and NS samples, Table S2), with the published cell-type annotations retained [60]. The processed counts were normalized using Seurat, 2000 highly variable genes were selected, and PCA and UMAP were performed using the first 30 principal components. Expression differences of the target genes across cell types were subsequently analyzed.

### 4.10. Molecular Docking

Protein–ligand docking was carried out using the standard workflow of the AutoDock suite (AutoDock Vina 1.1.2 and AutoDockTools 1.5.7) [61]. High-resolution protein structures were retrieved from the Protein Data Bank (PDB). For RECK, the full-length AlphaFold model was used because no complete experimental human structure was available. Water molecules and initial ligands were removed using PyMOL 3.1.6.1, while functionally relevant ions or cofactors were retained where appropriate. The protein receptors were processed in AutoDockTools, including polar hydrogen addition and charge assignment. The 3D structures of the ligands were prepared using OpenBabel 3.1.1. Docking calculations were conducted with AutoDock Vina with results visualized by PyMOL and Discovery Studio. Redocking was also performed for targets with suitable co-crystallized ligands using the same docking procedure, and heavy-atom RMSD values were calculated to evaluate the reliability of the docking workflow. Structure identifiers and docking parameters are provided in Table S3.

### 4.11. Molecular Dynamics (MD) Simulation

Exploratory MD simulations were performed in GROMACS 2025.3 following a standard procedure [62]. Ligands were described with the GAFF force field (RESP charges from Gaussian 16) while proteins were modeled with the AMBER14SB force field and solvated using the TIP3P water model. The system was then neutralized to 150 mM NaCl. Following energy minimization, 100-ps NVT equilibration at 300 K and 100-ps NPT equilibration at 300 K and 1 bar were performed. Temperature was controlled using the velocity-rescaling thermostat, and pressure was maintained using the Parrinello–Rahman barostat. A single 100-ns production trajectory was then generated for each complex using a 2-fs time step. Structural stability was assessed by root mean square deviation (RMSD), radius of gyration (Rg), and root mean square fluctuation (RMSF), while binding stability was monitored via the solvent-accessible surface area (SASA) and the hydrogen-bond dynamics. Finally, the binding free energy (ΔGbind) was calculated by MM-PBSA using snapshots extracted from the equilibrated 90–100 ns portion of each trajectory at 1-ns intervals. The entropy contribution was estimated using the interaction entropy (IE) method based on all snapshots included in the MM-PBSA analysis. Free energy landscapes (FELs) were generated using Rg and RMSD to evaluate the thermodynamic stability.

### 4.12. HPA Database Analysis

We examined MMP9 protein levels in normal skin and cSCC tissues as supportive protein-level evidence by analyzing immunohistochemical data available in the HPA database. One normal-skin specimen and one cSCC specimen were selected for qualitative illustration.

### 4.13. Cell Culture, Viability, and Proliferation Assays

The human cSCC cell line A431 and murine fibroblast cell line NIH/3T3 were purchased from Meisen (Zhejiang, China). The murine cSCC cell line XL50 was previously established in our laboratory from UV-induced SKH-1 mouse cSCC and deposited as CCTCC No. C201827. All cells were cultured in DMEM containing 10% FBS and 1% penicillin–streptomycin (100 U/mL penicillin, 100 μg/mL streptomycin) at 37 °C with 5% CO_2_ in a humidified incubator. 6PPDQ (TargetMol, T78474; CAS 2754428-18-5; ≥98% purity) was dissolved in DMSO at 10 mg/mL, stored protected from light at −80 °C in aliquots, and thawed once. Fresh DMSO stocks were diluted 1:1000 in culture medium to yield 0.1% DMSO in all exposure and matched vehicle-control groups. Proliferation of A431 cells was assessed via the CCK-8 assay (Meilunbio, Dalian, China) following 6PPDQ exposure at concentrations of 0, 20, 100, 500, 2500, 5000, 10,000, 20,000, and 40,000 ng/L for 24 h. All concentrations were nominal. Additionally, EdU incorporation was evaluated in cells treated with 6PPDQ (0, 20, 200, and 2000 ng/L, corresponding to 0.067, 0.670, and 6.70 nM) using an EdU proliferation kit (Cat# CX003, CellorLab, Shanghai, China) and quantified via fluorescence microscopy. Three non-overlapping fields were analyzed in each of five independently treated wells per group.

### 4.14. Quantitative Real-Time PCR

Following 24-h exposure to 6PPDQ at 0, 20, 200, and 2000 ng/L, total RNA from A431 cells was extracted with TRIzol and then reverse-transcribed. *MMP9* mRNA levels were assessed by qRT-PCR. The primer sequences were as follows: *MMP9* forward, 5′-AGACCTGGGCAGATTCCAAAC-3′; *MMP9* reverse, 5′-CGGCAAGTCTTCCGAGTAGT-3′; *GAPDH* forward, 5′-GGAGCGAGATCCCTCCAAAAT-3′; *GAPDH* reverse, 5′-GGCTGTTGTCATACTTCTCATGG-3′. Relative *MMP9* mRNA expression was normalized to GAPDH and calculated using the 2^−ΔΔCt^ method.

### 4.15. Western Blot Analysis

We used our established Western blotting protocol [63]. Total proteins from A431 cells treated with 6PPDQ (0, 20, 200, and 2000 ng/L) for 24 h were extracted and subjected to immunoblotting. The primary antibodies used were PI3K p110α (CST, #4249), p-AKT (Ser473; CST, #9271), pan-AKT (CST, #4691), MMP9 (Abcam, ab76003) and Actin (CST, #4967). Band intensities were quantified using ImageJ 1.53t. p-AKT was normalized to total AKT, whereas PI3K p110α and MMP9 were normalized to β-actin. All values are expressed relative to the vehicle-control mean.

### 4.16. Pharmacological AKT Inhibition

To evaluate the involvement of AKT signaling in the effects of 6PPDQ, A431 cells were pretreated with the AKT inhibitor MK-2206 (1 μM; MCE, HY-108232) for 1 h, followed by co-treatment with 6PPDQ (2000 ng/L) for 24 h. Cells were assigned to control, 6PPDQ, MK-2206, and MK-2206 plus 6PPDQ groups. Cell viability was assessed using the CCK-8 assay, and p-AKT, total AKT, and MMP9 protein levels were evaluated by Western blotting as described above.

### 4.17. siRNA Transfection

A431 cells were transfected with two *MMP9* siRNAs or control siRNA (si-NC) using EZ Trans siRNA Transfection Reagent (AC04L051, OBiO Technology, Shanghai, China). The sense sequences were 5′-GCUGCUUCUCCAGAAGCAA-3′ for si-MMP9-1, 5′-GCGAGAGACUCUACACCCA-3′ for si-MMP9-2, and 5′-UUCUCCGAACGUGUCACGU-3′ for si-NC. Knockdown efficiency was assessed by Western blotting 48 h after transfection.

### 4.18. Wound-Healing and Invasion Assays

For wound-healing assays, A431 cells were transfected with si-MMP9 or si-NC for 24 h, scratched with a sterile pipette tip, and then cultured in DMEM containing 1% FBS with or without 6PPDQ (2000 ng/L) for an additional 24 h. Images were acquired at 0 and 24 h, and wound closure was quantified relative to the initial wound area.

For invasion assays, cells were transfected for 24 h, harvested, and seeded into Transwell inserts pre-coated with 50 μL of 1 mg/mL Matrigel (Yeasen, Shanghai, China, 40183) at 6 × 10^4^ cells/insert. The upper chamber contained serum-free medium with or without 6PPDQ (2000 ng/L), and the lower chamber contained medium with 10% FBS. After 24 h, invaded cells were fixed, stained, imaged, and quantified.

### 4.19. Gelatin Zymography

A431 cells were exposed to 6PPDQ (0, 20, 200, or 2000 ng/L) in serum-free medium for 24 h. Conditioned media were centrifuged, concentrated using 30-kDa molecular-weight-cutoff filters (Millipore, Burlington, MA, USA, UFC803096), and loaded at volumes normalized to cell count [64]. Samples were mixed with non-reducing loading buffer and analyzed using a gelatin zymography kit (RTD6143; Zhongke Ruitai, Beijing, China). Gels were renatured, developed at 37 °C for 24 h, stained with FastBlue, and imaged using a ChemiDoc Imaging System (Bio-Rad, Hercules, CA, USA) in Coomassie Blue mode. The 92-kDa pro-MMP-9 band was quantified using ImageJ and expressed relative to the vehicle control.

### 4.20. Animal Models

All animal procedures received approval from the Institutional Animal Care and Use Committee of Shanghai Skin Disease Hospital (Ethics number: 2025-64). We complied with institutional and national standards for laboratory animal care in all experiments and reported them following the ARRIVE guidelines. Female immunocompetent SKH-1 mice (8 weeks old, 18–22 g) were obtained from the Shanghai Public Health Center and acclimatized for 7 days before the experiments. All experiments were performed in an approved animal facility (SYXK (Hu) 2020-0002), where mice were housed under SPF conditions (20–24 °C, 40–60% humidity, 12-h light/dark cycle) with free access to standard chow and water.

Following our previously reported implanted cSCC model, XL50 cells (5 × 10^6^) were mixed with NIH/3T3 fibroblasts (1 × 10^6^) in PBS and co-injected to provide stromal support for tumor growth [65]. For tumor cell implantation, mice were anesthetized with isoflurane (3–4% for induction, 1–2% for maintenance). The depth of anesthesia was monitored based on the respiratory pattern, absence of voluntary movement, and lack of response to toe pinch. Following previous mouse exposure studies using the same low-/high-dose range and intraperitoneal route, 6PPDQ was dissolved in sterile injectable olive oil at nominal concentrations of 0.04 or 0.4 mg/mL under light-protected conditions and mixed until visually homogeneous. The group size (*n* = 5) was selected based on previous studies [66,67]. The three treatment groups were vehicle control, 0.4 mg/kg 6PPDQ, and 4 mg/kg 6PPDQ [24]. All mice received intraperitoneal injections at 200 μL per mouse, with vehicle-control mice receiving sterile injectable olive oil alone. This route was selected to ensure controlled systemic exposure and reproducible dose delivery. Topical administration was not used because quantitative skin dosimetry and dermal dosing protocols for 6PPDQ remain insufficiently established.

Tumor cells were implanted on day 0. After confirmation of tumor establishment on day 7, mice were randomized to the three treatment groups and dosed every 3 days (days 7, 10, 13, 16, and 19). Tumor volumes were measured every 3 days from day 7 to day 21 after inoculation and calculated as (length × width^2^)/2. On day 21, we deeply anesthetized the mice with 4–5% isoflurane. After confirmation of deep anesthesia by the absence of voluntary movement and toe-pinch response, cervical dislocation was performed. Tumors were excised, weighed, and photographed. Tumor measurements and subsequent outcome assessments were performed by investigators blinded to group allocation.

### 4.21. Histological, IHC, and IF Evaluation

Excised tumors were fixed in 10% formalin, paraffin-embedded, and sectioned at 5 µm for H&E, IHC and IF staining. After deparaffinization, rehydration, antigen retrieval, endogenous peroxidase blocking, and BSA blocking, sections were incubated overnight with anti-Ki67 (Servicebio, Wuhan, China, GB111499, 1:1000) and anti-MMP9 (Servicebio, GB150096, 1:1000). Immunoreactivity was detected with an HRP-polymer secondary antibody (Servicebio, G1302) and DAB substrate (Servicebio, G1212), followed by hematoxylin counterstaining (Servicebio, G1004). Sections were dehydrated, mounted, and scanned. The percentage of Ki67-positive cells and MMP9 IHC scores were quantified for statistical analysis. For IF staining, sections were incubated with phospho-AKT (Ser473; CST, #9271, 1:2000), MMP9 (Servicebio, GB150096, 1:1000), and F4/80 (Servicebio, GB113373, 1:1000), followed by fluorescent secondary antibodies and DAPI counterstaining. p-AKT- and F4/80-positive area were quantified relative to the tumor area. Five non-overlapping fields per mouse were averaged, and each mouse was treated as one biological replicate (*n* = 5 mice/group).

### 4.22. Statistical Analysis

Data are presented as mean ± SD and all analyses were conducted in GraphPad Prism 10.4.0. Normality and homogeneity of variances were assessed using the Shapiro–Wilk and Brown–Forsythe tests, respectively. For single-factor multiple-group comparisons, one-way ANOVA with Dunnett’s post hoc test was used. For two-factor experiments, two-way ANOVA followed by Šídák’s multiple-comparisons test was used. Tumor growth curves were evaluated by two-way repeated-measures ANOVA with Geisser–Greenhouse correction followed by Dunnett’s test. Statistical significance was set at *p* < 0.05.

## 5. Conclusions

In conclusion, the study provides initial evidence for a potential mechanistic link between the emerging environmental contaminants 6PPD/6PPDQ and cSCC. Through a network toxicology framework combined with machine learning and multi-omics analysis, we identified a cSCC-associated six-gene candidate signature within the predicted 6PPD/6PPDQ–disease overlap and prioritized *MMP9* for experimental validation. Crucially, our experiments showed that 6PPDQ significantly accelerates cSCC cell proliferation and tumor growth, with the PI3K-Akt/MMP9 axis functionally implicated. These findings provide candidate molecular targets for future mechanistic validation and environmental risk assessment.

## Figures and Tables

**Table 1:** The binding free energy (ΔGbind) of 6PPD/6PPDQ with MMP9 (kJ/mol).

Complex	ΔE_vdW_	ΔE_ele_	ΔGgas	ΔG_PB_	ΔG_SA_	ΔG_solv_	ΔH	−TΔS	ΔG_bind_
6PPD-MMP9	−243.87	−2.81	−246.68	47.01	−18.99	28.02	−218.67	4.67	−213.98
6PPDQ-MMP9	−248.16	−7.99	−256.16	63.85	−19.99	43.86	−212.30	13.56	−198.74

ΔG_bind_ = ΔG_gas_ + ΔG_solv_ − TΔS. ΔG_gas_ is the total gas-phase energy (ΔE_vdW_ + ΔE_ele_). ΔG_solv_ is the total solvation energy (ΔG_PB_ + ΔG_SA_). ΔE_vdW_ denotes the van der Waals interaction, ΔE_ele_ is the electrostatic interaction, ΔG_PB_ is the polar solvation energy, and ΔG_SA_ is the non-polar solvation energy.

## Data Availability

Dataset available on request from the authors.

## Supplementary Materials

The following supporting information can be downloaded at https://www.mdpi.com/article/10.3390/ijms27188304/s1.
