# Supplementary material for: 6PPDQ Promotes Cutaneous Squamous Cell Carcinoma Growth with PI3K-Akt/MMP9 Activation: Evidence from Integrated Network Toxicology and Experimental Investigation
Source: Int J Mol Sci. 2026 Sep 18;27(18):8304. doi: 10.3390/ijms27188304 (PMC13607494; doi:10.3390/ijms27188304)
Supplement: Supplementary file 1 [file ijms-27-08304-s001.zip › Table S1.pdf]

Study	Accession in metadata	Final metadata n	NS	cSCC	Other sample categories in metadata	Library type	Present analysis	NS+cSCC used
Bailey_2023	PRJNA844527	109	26	66	AK 14; KA 3	ISR	Validation cohort 1	92
Chitsazzadeh_2016	GSE84293	26	7	9	AK 10	ISF, IU	Validation cohort 2	16
Cho_2018	GSE111582	18	10	8	—	ISR	Validation cohort 2	18
Hoang_2017	E-MTAB-5678	29	4	5	AK 13; IEC 7	ISR, IU	Training	9
Lee_2018	SRP126169	6	3	3	—	IU	Training	6
Li_2020	PRJNA556128	12	5	7	—	IU	Training	12
Lukowski_2018	E-MTAB-6430	25	0	3	AK 1; AK/IEC 8; AK/IEC/SCC 3; IEC 9; BCC 1	IU	Training	3
Mahapatra_2020	GSE139505	16	7	9	—	ISR	Training	16
Srivastava_2022	GSE191334	24	8	8	AK 8	ISR	Training	16
Vandiver_2021	GSE111354	22	3	19	—	IU	Validation cohort 2	22
Veenstra_2023	GSE235556	20	0	10	KA 10	ISR	Training	10
**Total**	—	**307†**	**73**	**147**	—	—	—	**220**

†The metadata file contains one BCC sample in addition to the 306 samples reported for the cSCC-progression cohort; this BCC sample was not used in the present cSCC-versus-normal analysis.
