# Supplementary material for: 6PPDQ Promotes Cutaneous Squamous Cell Carcinoma Growth with PI3K-Akt/MMP9 Activation: Evidence from Integrated Network Toxicology and Experimental Investigation
Source: Int J Mol Sci. 2026 Sep 18;27(18):8304. doi: 10.3390/ijms27188304 (PMC13607494; doi:10.3390/ijms27188304)
Supplement: Supplementary file 1 [file ijms-27-08304-s001.zip › Table S2.pdf]

Supplementary Table S2. Summary of donor representation and cell numbers in the GSE144236 scRNA-seq dataset

Donor	Normal	Tumor	Total
P1	971	1411	2382
P10	2204	2365	4569
P2	4088	2736	6824
P3	1296	446	1742
P4	229	7727	7956
P5	990	1504	2494
P6	4475	2185	6660
P7	2199	3312	5511
P8	587	2920	3507
P9	4826	1693	6519

Note: The table summarizes the numbers of processed cells contributed by each donor under normal-skin and cSCC conditions. The dataset comprised 48,164 cells from 10 donors with paired normal-skin and cSCC samples. Detailed donor-, condition-, and cell-type-specific cell counts are provided separately as an accompanying supplementary CSV file.
