# Supplementary material for: 6PPDQ Promotes Cutaneous Squamous Cell Carcinoma Growth with PI3K-Akt/MMP9 Activation: Evidence from Integrated Network Toxicology and Experimental Investigation
Source: Int J Mol Sci. 2026 Sep 18;27(18):8304. doi: 10.3390/ijms27188304 (PMC13607494; doi:10.3390/ijms27188304)
Supplement: Supplementary file 1 [file ijms-27-08304-s001.zip › Table S3.pdf]

Target	PDB ID	Native ligand	Retained ions / cofactors	Grid center (x,y,z), Å	Grid size (x,y,z), Å	Exhaustiveness	Redocking score (kcal/mol)	Heavy-atom RMSD (Å)
MMP1	1HFC	PLH	Zn^{2+}; Ca^{2+}	14.118, 29.748, 23.217	42, 46, 44	20	−7.3	0.448
MMP9	6ESM	B9Z	Zn^{2+}; Ca^{2+}	−0.057, 43.026, 14.861	42, 34, 44	20	−10.8	0.104
GSTP1	3GUS	N11	GSH	18.273, −0.078, 20.487	50, 42, 54	20	−5.5	1.572
LGALS3	5EXO	5SY	None	13.235, 0.439, 6.552	40, 46, 40	20	−6.5	0.573
FOXM1	3G73	—	None	−44.717, 9.724, −31.730	38, 34, 40	20	N/A	N/A
RECK	AF-O95980-F1†	—	N/A	−7.240, 2.098, 4.187	96, 70,110	20	N/A	N/A

† For RECK, the full-length AlphaFold model was used because no complete experimental human structure was available.
