# Supplementary material for: 6PPDQ Promotes Cutaneous Squamous Cell Carcinoma Growth with PI3K-Akt/MMP9 Activation: Evidence from Integrated Network Toxicology and Experimental Investigation
Source: Int J Mol Sci. 2026 Sep 18;27(18):8304. doi: 10.3390/ijms27188304 (PMC13607494; doi:10.3390/ijms27188304)
Supplement: Supplementary file 1 [file ijms-27-08304-s001.zip › Table S4.pdf]

Abbreviations

The following abbreviations are used in this manuscript:

6PPD	N-(1,3-dimethylbutyl)-N′-phenyl-p-phenylenediamine
6PPDQ	6PPD-quinone
AKT	AKT serine/threonine kinase (protein kinase B)
ANOVA	analysis of variance
ARRIVE	Animal Research: Reporting of In Vivo Experiments
ASK1	apoptosis signal-regulating kinase 1
AUC	area under the curve
BP	biological process
BSA	bovine serum albumin
CC	cellular component
CCK-8	Cell Counting Kit-8
CCTCC	China Center for Type Culture Collection
CIBERSORT	Cell-type Identification By Estimating Relative Subsets Of RNA Transcripts
CRISPR-Cas9	clustered regularly interspaced short palindromic repeats/CRISPR-associated protein 9
CSC	cancer stem cell
cSCC	cutaneous squamous cell carcinoma
CSF	cerebrospinal fluid
CTD	Comparative Toxicogenomics Database
DAB	3,3′-diaminobenzidine
DEG	differentially expressed gene
DMBA	7,12-dimethylbenz[a]anthracene
DMEM	Dulbecco’s modified Eagle medium
ECM	extracellular matrix
EdU	5-ethynyl-2′-deoxyuridine
EMT	epithelial-to-mesenchymal transition
ERK	extracellular signal-regulated kinase
FBS	fetal bovine serum
FC	fold change
FEL	free energy landscape
FOXM1	forkhead box M1
FOXO1	forkhead box O1
GAFF	General AMBER Force Field
GAPDH	glyceraldehyde-3-phosphate dehydrogenase
GEO	Gene Expression Omnibus
glmBoost	generalized linear model boosting
GO	Gene Ontology
GS	gene significance
GSTP1	glutathione S-transferase P1
H&E	hematoxylin and eosin

HNSCC	head and neck squamous cell carcinoma
HPA	Human Protein Atlas
HRP	horseradish peroxidase
HTR2A	5-hydroxytryptamine receptor 2A
IE	interaction entropy
IF	immunofluorescence
IHC	immunohistochemistry
IL-17	interleukin-17
JNK	c-Jun N-terminal kinase
KEGG	Kyoto Encyclopedia of Genes and Genomes
LASSO	least absolute shrinkage and selection operator
LDA	linear discriminant analysis
LGALS3	galectin-3
MAPK	mitogen-activated protein kinase
MD	molecular dynamics
MDSC	myeloid-derived suppressor cell
MF	molecular function
MM	module membership
MM-PBSA	molecular mechanics Poisson–Boltzmann surface area
MMP	matrix metalloproteinase
MMP1	matrix metalloproteinase 1
MMP2	matrix metalloproteinase 2
MMP9	matrix metalloproteinase 9
mRNA	messenger RNA
NPT	constant number of particles, pressure, and temperature ensemble
NRF2	nuclear factor erythroid 2-related factor 2
NS	normal skin
NVT	constant number of particles, volume, and temperature ensemble
OMIM	Online Mendelian Inheritance in Man
p-AKT	phosphorylated AKT
PAH	polycyclic aromatic hydrocarbons
PBS	phosphate-buffered saline
PCA	principal component analysis
PDB	Protein Data Bank
PI3K	phosphoinositide 3-kinase
plsRglm	partial least squares regression for generalized linear models
PM	particulate matter
PPI	protein–protein interaction
qRT-PCR	quantitative reverse-transcription polymerase chain reaction
RB1	retinoblastoma protein 1
RECK	reversion-inducing cysteine-rich protein with Kazal motifs
RESP	restrained electrostatic potential

Rg	radius of gyration
RMSD	root mean square deviation
RMSF	root mean square fluctuation
ROC	receiver operating characteristic
ROS	reactive oxygen species
SASA	solvent-accessible surface area
scRNA-seq	single-cell RNA sequencing
SD	standard deviation
SEA	Similarity Ensemble Approach
SHAP	SHapley Additive exPlanations
SMILES	simplified molecular input line entry system
SPF	specific pathogen-free
Stepglm	stepwise generalized linear model
STRING	Search Tool for the Retrieval of Interacting Genes/Proteins
SVM	support vector machine
TIP3P	three-point transferable intermolecular potential water model
TNF	tumor necrosis factor
TOM	topological overlap matrix
TPA	12-O-tetradecanoylphorbol-13-acetate
TRAF2	tumor necrosis factor receptor-associated factor 2
TSK	tumor-specific keratinocyte
UMAP	uniform manifold approximation and projection
UV	ultraviolet
VEGF-A	vascular endothelial growth factor A
VST	variance-stabilizing transformation
WGCNA	weighted gene co-expression network analysis
XGBoost	extreme gradient boosting
